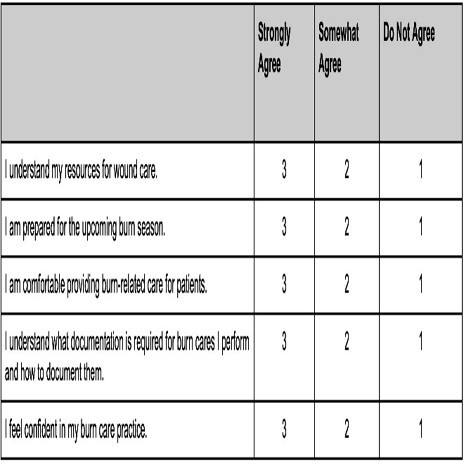# 556 Certified Nurse Assistant Burn-Specific Skills Day: A Training Paradigm for Critical Members of Burn Teams

**DOI:** 10.1093/jbcr/irae036.190

**Published:** 2024-04-17

**Authors:** Micayla Kotarski, Kathleen M Ewanowski, Danielle Westbroek, Callie M Thompson

**Affiliations:** University of Utah Burn Center, Bountiful, UT; University of Utah Health Burn Center, Salt Lake City, UT; University of Utah, Bountiful, UT; University of Utah Health, Salt Lake City, UT; University of Utah Burn Center, Bountiful, UT; University of Utah Health Burn Center, Salt Lake City, UT; University of Utah, Bountiful, UT; University of Utah Health, Salt Lake City, UT; University of Utah Burn Center, Bountiful, UT; University of Utah Health Burn Center, Salt Lake City, UT; University of Utah, Bountiful, UT; University of Utah Health, Salt Lake City, UT; University of Utah Burn Center, Bountiful, UT; University of Utah Health Burn Center, Salt Lake City, UT; University of Utah, Bountiful, UT; University of Utah Health, Salt Lake City, UT

## Abstract

**Introduction:**

High turnover rates among Certified Nursing Assistants (CNAs) in acute care settings disrupt the continuity of care for patients and increase financial burden on healthcare institutions. Burn care is uniquely demanding and emotionally taxing. Our center utilizes “Burn-Specific Skills Days” (BSSDs) as a creative approach to enhance job satisfaction, professional development, and the longevity of our CNAs. BSSDs are designed to address the unique needs and challenges faced by CNAs in their daily roles on the Burn unit, ultimately contributing to their retention and increasing the overall quality of their patient care.

The key components of BSSD include targeted training for summer and winter burn care, peer-provided support and recognition programs, and how BSSDs encourage the commitment of CNAs to the burn unit and their organization.

Through feedback from CNAs in biannual BSSDs, this effort aims to shed light on the potential of BSSD to enhance the sustainability of CNAs in the burn unit. Ultimately, BSSDs emphasize the importance of investing in the professional development and wellbeing of CNAs on the burn unit as a means of ensuring continuity of high-quality patient care, thus improving patient outcomes.

**Methods:**

We implement two BSSDs at different times of the year to address season-specific injuries encountered in the Burn ICU. Spring BSSDs focus on burn injuries commonly associated with summer and fall, including those caused by campfires, hot coals, and flash burns. Alternatively, fall BSSDs provide education for injuries more prevalent during winter and spring, including necrotizing fasciitis, frostbite, and inhalation injuries. Additionally, both BSSDs incorporate a large TBSA burn admission simulation which reinforces established roles CNAs perform during these events.

A pre-education survey is given prior to the BSSD. A 15 minute debrief and peer-provided feedback session close each BSSD. Within a week, a 10 question survey is sent so more feedback can be collected after reflection time.

**Results:**

Initial support and excitement for BSSDs has been high. We anticipate 100% participation from our Burn CNAs. Preliminary results show increased confidence in burn-specific skills, individual acknowledgment, team cohesion, and job satisfaction.

**Conclusions:**

We have described a novel program for skill-building and retention among Burn CNAs, a critical piece of the burn care team that has been historically left out of such efforts. Much like competence building programs for nursing, we anticipate that BSSDs will play an important role in improving competence, confidence, and job satisfaction of Burn CNAs. This will in turn lead to better patient care, reduced turnover rates, and a more positive work environment.

**Applicability of Research to Practice:**

This biannual training functions to maintain competency and confidence of CNAs on best practice for burn care. Such programs can easily be adapted and scaled for use at all ABA-verified Burn Centers.